# Correction: How much (ATP) does it cost to build a trypanosome? A theoretical study on the quantity of ATP needed to maintain and duplicate a bloodstream-form *Trypanosoma brucei* cell

**DOI:** 10.1371/journal.ppat.1011617

**Published:** 2023-08-30

**Authors:** Janaina F. Nascimento, Rodolpho O. O. Souza, Mayke B. Alencar, Sabrina Marsiccobetre, Ana M. Murillo, Flávia S. Damasceno, Richard B. M. M. Girard, Letícia Marchese, Luis A. Luévano-Martinez, Renan W. Achjian, Jurgen R. Haanstra, Paul A. M. Michels, Ariel M. Silber

In the Abstract, the seventh sentence is incorrect. The correct sentence reads: Total biomass production (which involves biomass maintenance and duplication) accounts for ~63% of the total energy budget, while other ATP-dependent processes account for the remaining ~37% of the ATP consumption, with translation being the most expensive process.

In the penultimate sentence in the final paragraph of the Introduction, the value “62%” should be “63%.”

In the Results section, the fourth paragraph should be preceded by the subtitle “The cost of genome duplication.”

The first sentence of the sub-section “Energy cost of sugar nucleotides used in the synthesis of the VSG coat” is incorrect. The correct sentence reads: In the BSF of *T*. *brucei*, the major surface protein is the VSG, which is highly glycosylated and linked to the membrane by GPI anchors.

The seventh sentence of the sub-section “ATP requirement for transmembrane transport” is incorrect. The correct sentence reads: It is worth mentioning that Stouthamer did not consider the costs of taking up glucose, which could be relevant for many prokaryotes but not for BSF *T*. *brucei* where glucose transport happens by facilitated diffusion [115,116].

The penultimate sentence in the sub-section “How much ATP hydrolysis is required to maintain the mitochondrial inner membrane potential (ΔΨm)?” is incorrect. The correct sentence reads: We hypothesize that this mitochondrial substrate-level phosphorylation system is the main source of intramitochondrial ATP, and it can provide sufficient ATP to maintain the ΔΨ_m_ [22,157], despite its relatively low capacity for producing ATP [10].

In Table 11, the BSF *Trypanosoma brucei* “Total” value should be 6.00 x 10^11^.

The seventeenth sentence of the third Discussion paragraph is incorrect. The correct sentence reads: As we considered 2 ATP molecules being spent per base polymerized, an average transcript length of 2,800 nt, and an average RNA synthesis of 1.2 RNAs/h (estimated in [51]) the estimated ATP expenditure for nuclear transcription is ~2.9 x 10^7^ ATP molecules (0.5% of the total ATP expenditure for the completion of a cell cycle) (Fig 1, S5 Table).

The fourth sentence of the fourth Discussion paragraph is incorrect. The correct sentence reads: Therefore, taken together, translation and protein turnover demand 59.6% of the ATP budget (Table 13).

The penultimate sentence of the fourth Discussion paragraph is incorrect. The correct sentence reads: These calculations do not include the cost of the synthesis of sugar nucleotides used for the glycosylation of surface proteins (mostly VSGs) and anchoring.

The ninth sentence of the seventh Discussion paragraph is incorrect. The correct sentence reads: To estimate the total percentage of the budget used for flagellar beating, we considered the highest value obtained, which resulted in the consumption of 9.5% of ATP produced (Table 13).
