# Supplementary material for: How much (ATP) does it cost to build a trypanosome? A theoretical study on the quantity of ATP needed to maintain and duplicate a bloodstream-form Trypanosoma brucei cell
Source: PLoS Pathog. 2023 Jul 27;19(7):e1011522. doi: 10.1371/journal.ppat.1011522 (PMC10409291; doi:10.1371/journal.ppat.1011522)
Supplement: S1 Table — (PDF) [file ppat.1011522.s001.pdf]

Supplementary Table S1. Composition of HMI-9 and CMM

HMI-9

**Components**	**mg/L**
$CaCl_2$	165
KCl	330
KNO_3	0.076
$MgSO_4$	98
NaCl	4500
$NaHCO_3$	3020
$NaH_2PO_4.H_2O$	125
$Na_2SeO_3.5H_2O$	0.017
Phenol red	15
HEPES	5960
Mercaptoethanol	15
Bathocuproine disulfonate.Na_2	28
Glucose	4500
L-Alanine	25
L-Arginine.HCl	84
L-Asparagine	25
L-Aspartate	30
L-Cysteine	182
Cystine	91
L-Glutamate	75
L-Glutamine	584
L-Histidine.HCl.H_2O	42
L-Isoleucine	105
L-Leucine	105
L-Lysine.HCl	146
L-Methionine	30
L-Phenylalanine	66
L-Proline	40
L-Serine	42
L-Threonine	95
L-Tryptophan	16
L-Tyrosine	104
L-Valine	94
B12	0.013
Biotin	0.013
D-C pantothenate	4
Choline chloride	4
Niacinamide	4
Pyridoxal.HCl	4
Riboflavin	0.04
Thiamine.HCl	4
Pyruvate.Na	114
Hypoxanthine	136
Uracil	10
Cytosine	10
Serum	10%

References: [1]; http://tryps.rockefeller.edu/trypsru2_culture_media_compositions.html

CMM

**Components**	**mg/L**
D-Glucose	1800
L-Glutamine	146
L-Cysteine	121
NaCl	4500
$CaCl_2 \cdot 2H_2O$	219
KCl	330
$MgSO_4 \cdot 7H_2O$	200
$NaHCO_3$	3020
HEPES	5960
Phenol red	15
Bathocuproinedisulfonic acid	28
Mercaptoethanol	192.15 µl
FCS Gold (PAA)	10%, 100 ml

Reference: [2]

References

1. Hirumi H, Hirumi K. Continuous cultivation of *Trypanosoma brucei* bloodstream forms in a medium containing a low concentration of serum protein without feeder cell layers. J Parasitol 1989; 75:985–9.

2. Creek DJ, Nijagal B, Kim DH, Rojas F, Matthews KR, Barrett MP. Metabolomics guides rational development of a simplified cell culture medium for drug screening against *Trypanosoma brucei*. Antimicrob Agents Chemother. 2013; 57:2768–2779. doi:10.1128/AAC.00044-13
