# Supplementary material for: How much (ATP) does it cost to build a trypanosome? A theoretical study on the quantity of ATP needed to maintain and duplicate a bloodstream-form Trypanosoma brucei cell
Source: PLoS Pathog. 2023 Jul 27;19(7):e1011522. doi: 10.1371/journal.ppat.1011522 (PMC10409291; doi:10.1371/journal.ppat.1011522)
Supplement: S2 Table — (PDF) [file ppat.1011522.s002.pdf]

Supplementary Table S2. Reactions for synthesis of dNTPs

dCTP from Gln (costs 12 ATPs)

1 2 ATP + L-glutamine + hydrogen carbonate + H_2O = 2 ADP + phosphate + L-glutamate + carbamoyl phosphate
2 carbamoyl phosphate + L-aspartate = phosphate + N-carbamoyl-L-aspartate
3 N-Carbamoyl-L-aspartate <=> (S)-Dihydroorotate + H_2O
4 (S)-Dihydroorotate + Fumarate (-2 ATPs)* <=> Orotate + Succinate
5 Orotate + 5-phospho-alpha-D-ribose 1-diphosphate (PRPP) (+3 ATPs)* = Orotidine 5'-phosphate + diphosphate
6 Orotidine 5'-phosphate <=> UMP + CO_2
7 UMP + Orthophosphate <=> UDP + H_2O
8 ATP + UDP = ADP + UTP
9 ATP + UTP + Ammonia <=> ADP + Orthophosphate + CTP
10 ADP + CTP = ATP + CDP
11 Thioredoxin (+6 ATPs)* + CDP <=> dCDP + Thioredoxin disulfide + H_2O (Trypanothione – [1])
12 ATP + dCDP = ADP + dCTP

dTTP from Gln (costs 6 ATPs)

1 2 ATP + L-glutamine + hydrogen carbonate + H_2O = 2 ADP + phosphate + L-glutamate + carbamoyl phosphate
2 Carbamoyl phosphate + L-aspartate = phosphate + N-carbamoyl-L-aspartate
3 N-Carbamoyl-L-aspartate <=> (S)-Dihydroorotate + H_2O
4 (S)-Dihydroorotate + Fumarate (-2 ATPs)* <=> Orotate + Succinate
5 Orotate + 5-phospho-alpha-D-ribose 1-diphosphate (PRPP) (+3 ATPs)* = orotidine 5'-phosphate + diphosphate
6 Orotidine 5'-phosphate <=> UMP + CO_2
7 UMP + Diphosphate <=> Uracil + 5-Phospho-alpha-D-ribose 1-diphosphate
8 Uracil + 2-Deoxy-D-ribose 1-phosphate (0 ATP)* <=> Deoxyuridine + Orthophosphate
9 ATP + Deoxyuridine <=> ADP + dUMP
10 dUMP + 5,10-Methylenetetrahydrofolate (0 ATP)* <=> Dihydrofolate + dTMP
11 ATP + dTMP <=> ADP + dTDP
12 ATP + dTDP = ADP + dTTP

dATP from hypoxanthine (costs 11 ATPs)

1 hypoxanthine + 5-phospho-alpha-D-ribose 1-diphosphate (+3 ATPs)* = IMP + diphosphate
2 IMP + Ammonia <=> AMP + H2O
3 ATP + AMP <=> 2 ADP
4 Thioredoxin (+6 ATPs)* + ADP <=> dADP + Thioredoxin disulfide + H2O (Trypanothione – [1])
5 ATP + dADP <=> ADP + dATP

dGTP from hypoxanthine (costs 12 ATPs)

1 hypoxanthine + 5-phospho-alpha-D-ribose 1-diphosphate (+3 ATPs)* = IMP + diphosphate
2 IMP + NAD+ + H_2O <=> XMP + NADH + H+
3 ATP + XMP + Ammonia <=> AMP + Diphosphate + GMP
4 ATP + GMP <=> ADP + GDP
5 Thioredoxin (+6 ATPs)* + GDP <=> dGDP + Thioredoxin disulfide + H_2O (Trypanothione – [1])
6 ATP + dGDP <=> ADP + dGTP

*Reactions for synthesis of the precursors of dNTPS and rNTPs are described in S4 Table

References

1. Dormeyer M, Reckenfelderbäumer N, Lüdemann H, Krauth-Siegel RL. Trypanothione-dependent synthesis of deoxyribonucleotides by *Trypanosoma brucei* ribonucleotide reductase. Journal of Biological Chemistry. 2001; 276:10602–10606. doi:10.1074/jbc.M010352200
