# Supplementary material for: How much (ATP) does it cost to build a trypanosome? A theoretical study on the quantity of ATP needed to maintain and duplicate a bloodstream-form Trypanosoma brucei cell
Source: PLoS Pathog. 2023 Jul 27;19(7):e1011522. doi: 10.1371/journal.ppat.1011522 (PMC10409291; doi:10.1371/journal.ppat.1011522)
Supplement: S3 Table — (PDF) [file ppat.1011522.s003.pdf]

Supplementary Table S3. Reactions for synthesis of rNTPs

CTP from Gln (costs 5 ATPs)

1. 2 ATP + L-glutamine + hydrogencarbonate + H_2O = 2 ADP + phosphate + L-glutamate + carbamoyl phosphate
2. Carbamoyl phosphate + L-aspartate = phosphate + N-carbamoyl-L-aspartate
3. N-Carbamoyl-L-aspartate <=> (S)-Dihydroorotate + H_2O
4. (S)-Dihydroorotate + Fumarate (-2 ATP)* <=> Orotate + Succinate
5. Orotate + 5-phospho-alpha-D-ribose 1-diphosphate (PRPP) (+3 ATP)* = orotidine 5'-phosphate + diphosphate
6. Orotidine 5'-phosphate <=> UMP + CO_2
7. UMP + Orthophosphate <=> UDP + H_2O
8. ATP + UDP = ADP + UTP
9. ATP + UTP + Ammonia <=> ADP + Orthophosphate + CTP

UTP from Gln (costs 4 ATPs)

1. 2 ATP + L-glutamine + hydrogencarbonate + H_2O = 2 ADP + phosphate + L-glutamate + carbamoyl phosphate
2. carbamoyl phosphate + L-aspartate = phosphate + N-carbamoyl-L-aspartate
3. N-Carbamoyl-L-aspartate <=> (S)-Dihydroorotate + H_2O
4. (S)-Dihydroorotate + Fumarate (-2 ATPs)* <=> Orotate + Succinate
5. Orotate + 5-phospho-alpha-D-ribose 1-diphosphate (PRPP) (+3 ATPs)* = orotidine 5'-phosphate + diphosphate
6. Orotidine 5'-phosphate <=> UMP + CO_2
7. UMP + Orthophosphate <=> UDP + H_2O
8. ATP + UDP = ADP + UTP

ATP from hypoxanthine (costs 5 ATPs)

1. Hypoxanthine + 5-phospho-alpha-D-ribose 1-diphosphate (+3 ATPs)* = IMP + diphosphate
2. IMP + Ammonia <=> AMP + H_2O
3. ATP + AMP <=> 2 ADP
4. ATP + ADP <=> ADP + ATP

GTP from hypoxanthine (costs 6 ATPs)

1. Hypoxanthine + 5-phospho-alpha-D-ribose 1-diphosphate (+3 ATPs)* = IMP + diphosphate
2. IMP + NAD+ + H_2O <=> XMP + NADH + H+
3. ATP + XMP + Ammonia <=> AMP + Diphosphate + GMP
4. ATP + GMP <=> ADP + GDP
5. ATP + GDP <=> ADP + GTP

*Reactions for synthesis of the precursors of dNTPS and rNTPs are described in S4 Table
