# Supplementary material for: How much (ATP) does it cost to build a trypanosome? A theoretical study on the quantity of ATP needed to maintain and duplicate a bloodstream-form Trypanosoma brucei cell
Source: PLoS Pathog. 2023 Jul 27;19(7):e1011522. doi: 10.1371/journal.ppat.1011522 (PMC10409291; doi:10.1371/journal.ppat.1011522)
Supplement: S4 Table — (PDF) [file ppat.1011522.s004.pdf]

Supplementary Table S4. Reactions for synthesis of precursors of dNTPs and rNTPs

5-phospho-alpha-D-ribose 1-diphosphate (PRPP) from Glucose (costs 3 ATPs)
1 Glucose + ATP -> -> D-Frutose 6-phosphate
2 D-Fructose 6-phosphate + D-Glyceraldehyde 3-phosphate (+1 ATP) <=> D-Erythrose 4-phosphate + D-Xylulose 5-phosphate
3 D-Xylulose 5-phosphate <=> D-Ribulose 5-phosphate
4 D-Ribulose 5-phosphate <=> D-Ribose 5-phosphate
5 ATP + D-Ribose 5-phosphate <=> AMP + 5-Phospho-alpha-D-ribose 1-diphosphate

D-Glyceraldehyde 3-phosphate from glucose (costs 2 ATPs and produces 2 D-Glyceraldehyde 3-phoshate molecules)
1 Glucose + 2 ATP -> -> -> -> 2 D-Glyceraldehyde 3-phosphate

Fumarate from glucose (produces 4 ATPs and 2 fumarate molecules)
1 Glucose -> -> -> -> 2 Fumarate + 4 ATP

2-Deoxy-D-ribose 1-phosphate from glucose (costs 0 ATP)
1 Glucose + 2 ATP -> -> -> -> 2 D-Glyceraldehyde 3-phosphate
2 D-Glyceraldehyde 3-phosphate + Acetaldehyde (+2 ATPs) <=> 2-Deoxy-D-ribose 5-phosphate
3 2-Deoxy-D-ribose 5-phosphate <=> 2-Deoxy-D-ribose 1-phosphate

Acetaldehyde from glucose (produces 2 ATPs per pyruvate molecule)
1 Glucose -> -> -> -> 2 Pyruvate + 2 ATP
2 Pyruvate + CoA + NAD+ = acetyl-CoA + CO_2 + NADH + H+
3 AMP + Diphosphate + Acetyl-CoA <=> ATP + Acetate + CoA
4 Acetate + NADH + H+ <=> Acetaldehyde + NAD+ + H_2O

Trypanothione from Cys, Glu and Arg (costs 6 ATPs)
Step 1
1 ATP + L-Glutamate + L-Cysteine <=> ADP + Orthophosphate + gamma-L-Glutamyl-L-cysteine
2 2 ATP + 2 gamma-L-Glutamyl-L-cysteine + 2 Glycine <=> 2 ADP + 2 Orthophosphate + 2 Glutathione
Step 2
1 L-Arginine + H2O <=> L-Ornithine + Urea
2 L-Ornithine <=> Putrescine + CO2
3 S-Adenosylmethioninamine (+1 ATP) + Putrescine <=> 5'-Methylthioadenosine + Spermidine
4 ATP + Glutathione + Spermidine <=> ADP + Orthophosphate + Glutathionylspermidine
5 ATP + Glutathionylspermidine + Glutathione <=> ADP + Orthophosphate + Trypanothione

5,10-Methylenetetrahydrofolate from Glycine and Folate (costs 0 ATP)
1 Folate + 2 NADH + 2 H+ <=> Tetrahydrofolate + 2 NAD+
2 Glycine + Tetrahydrofolate + NAD+ <=> 5,10-Methylenetetrahydrofolate + Ammonia + CO_2 + NADH + H+

S-Adenosylmethioninamine from Methionine (costs 1 ATP)
1 ATP + L-Methionine + H_2O <=> Orthophosphate + Diphosphate + S-Adenosyl-L-methionine
2 S-Adenosyl-L-methionine + H+ <=> S-Adenosylmethioninamine + CO_2
