# Supplementary material for: How much (ATP) does it cost to build a trypanosome? A theoretical study on the quantity of ATP needed to maintain and duplicate a bloodstream-form Trypanosoma brucei cell
Source: PLoS Pathog. 2023 Jul 27;19(7):e1011522. doi: 10.1371/journal.ppat.1011522 (PMC10409291; doi:10.1371/journal.ppat.1011522)
Supplement: S5 Table — (PDF) [file ppat.1011522.s005.pdf]

Supplementary Table S5. Summary of ATP production and expenditure in BSF trypanosomes

Process	ATP per cell cycle per cell (x 10^6)	ATP demand (%)
Available from glucose	600000	...
Proteome duplication	200000	33.3
Protein turnover	158000	26.3
ATP requirement for transport (aa, K, P)	131000	21.8
Flagellar motility	57000	9.5
Lipids and sterols content	11900	2.0
Transcription (synthesis - nuclear)	5721	1.0
Transcription (maintenance - nuclear)	725,2	0.1
Transcription (synthesis - kDNA)	188	0.0
Transcription (maintenance - kDNA)	NA*	-
DNA replication (nuclear)	1493	0.2
DNA replication (kDNA)	75,9	0.0
Sugar nucleotides synthesis	1000	0.2
polyP synthesis and maintenance	29	0.0
Activation/recruitment of vesicles	0,114	0.0
ATP free remaining ATP	32867.8	5.5

*Not available due to the lack of data
