# Supplementary material for: How much (ATP) does it cost to build a trypanosome? A theoretical study on the quantity of ATP needed to maintain and duplicate a bloodstream-form Trypanosoma brucei cell
Source: PLoS Pathog. 2023 Jul 27;19(7):e1011522. doi: 10.1371/journal.ppat.1011522 (PMC10409291; doi:10.1371/journal.ppat.1011522)
Supplement: S6 Table — (PDF) [file ppat.1011522.s006.pdf]

Supplementary Table S6. Amino acids transport and metabolic pathways in *T. brucei*

		**Arginine**		
**Transport**	**Reference**	**Enzymes**	**Amino acid produced**	**Reference**
TbAAT5-3		Arginase (but it's not functional)	-	[2]
Km 3.6 ± 0.4 μM	[1]	Arginine kinase	-	[3]
~Vmax 13 pmol min^{-1} 10^{6} cells^{-1}				
		**Lysine**		
**Transport**	**Reference**	**Enzymes**		**Reference**
TbAAT16-1		-	-	
Km 4.3 ± 0.5 μM	[4]			
~Vmax 2.5 pmol min^{-1} 10^{6} cells^{-1}				
		**Aspartate**		
**Transport**	**Reference**	**Enzymes**	**Amino acid produced**	**Reference**
NI		ASAT	Glu	[5]
		AS	Asn	[6]
		**Asparagine**		
**Transport**	**Reference**	**Enzymes**	**Amino acid produced**	**Reference**
NI		-	-	
		**Glutamate**		
**Transport**	**Reference**	**Enzymes**	**Amino acid produced**	**Reference**
Km 158 ± 30 μM	[7]	GS (Tb927.7.4970)	Gln	NI
Vmax 30 ± 5 pmol s^{-1} 10^{8} cells^{-1}		ALAT	Ala	[8]
		**Glutamine**		
**Transport**	**Reference**	**Enzymes**	**Amino acid produced**	**Reference**
Not characterized yet	Damasceno, F. et al., unpublished data	KAT/GlnAT (only procyclic)	Ala	[9]
		**Cysteine**		
**Transport**	**Reference**	**Enzymes**	**Amino acid produced**	**Reference**
Km 0,4 μM	[10]	KAT/GlnAT (only procyclic)	-	[9]
Vmax 3 nmol min^{-1} 10^{7} cells^{-1}		???	Met	
		**Histidine**		
**Transport**	**Reference**	**Enzymes**	**Amino acid produced**	**Reference**
NI		-	-	
		**Alanine**		
**Transport**	**Reference**	**Enzymes**	**Amino acid produced**	**Reference**
Verified but not characterized	[11]	ALAT	Glu	[8]
		**Leucine**		
**Transport**	**Reference**	**Enzymes**	**Amino acid produced**	**Reference**
Verified but not characterized	[12]	-	-	
		**Isoleucine**		
**Transport**	**Reference**	**Enzymes**	**Amino acid produced**	**Reference**
NI.	[13]	-	-	
		**Valine**		
**Transport**	**Reference**	**Enzymes**	**Amino acid produced**	**Reference**
NI.		-	-	
		**Glycine**		
**Transport**	**Reference**	**Enzymes**	**Amino acid produced**	**Reference**
TbAAT	[4]	(SHMT) No putative genes	-	
		**Serine**		
**Transport**	**Reference**	**Enzymes**	**Amino acid produced**	**Reference**
TbAAT	[4]	(SHMT and 3-PG) No putative genes	-	
		**Methionine**		
**Transport**	**Reference**	**Enzymes/Putative gene**	**Amino acid produced**	**Reference**
K_M 32.8 μM and V_{max} 28.8 nmol min^{-1} (1×10^{8} cells)$^{-1}$	[14]			
TbAAT	[4]	Tb927.8.2610 / Tb927.1.1270	-	
		**Phenylalanine**		
**Transport**	**Reference**	**Enzymes**	**Amino acid produced**	**Reference**
TbAAT	[4]	No putative genes	-	
		**Tyrosine**		
**Transport**	**Reference**	**Enzymes**	**Amino acid produced**	**Reference**
TbAAT	[4]	No putative genes	-	
		**Tryptophan**		
**Transport**	**Reference**	**Enzymes**	**Amino acid produced**	**Reference**
TbAAT	[4]	No putative genes	-	
		**Threonine**		
**Transport**	**Reference**	**Enzymes**	**Amino acid produced**	**Reference**
TbAAT	[4]	(Aspartyl-B-P pathway) No putative genes	-	

NI = no information in the literature

References

1. Mathieu C, Macêdo JP, Hürlimann D, Wirdnam C, Haindrich AC, Grotemeyer MS, et al. Arginine and lysine transporters are essential for *Trypanosoma brucei*. PLoS One. 2017; 12:1–23. doi:10.1371/journal.pone.0168775
2. Hai Y, Kerkhoven EJ, Barrett MP, Christianson DW. Crystal structure of an arginase-like protein from *Trypanosoma brucei* that evolved without a binuclear manganese cluster. Biochemistry 2015; 54:458–471. doi:10.1021/bi501366a
3. Voncken F, Gao F, Wadforth C, Harley M, Colasante C. The phosphoarginine energy-buffering system of *Trypanosoma brucei* involves multiple arginine kinase isoforms with different subcellular locations. PLoS One. 2013; 8. doi:10.1371/journal.pone.0065908
4. Mathieu C, Salgado AG, Wirdnam C, Meier S, Grotemeyer MS, Inbar E, et al. *Trypanosoma brucei* eflornithine transporter AAT6 is a low-affinity low-selective transporter for neutral amino acids. Biochemical Journal. 2014; 463:9–18. doi:10.1042/BJ20140719
5. Marciano D, Llorente C, Maugeri DA, de la Fuente C, Opperdoes F, Cazzulo JJ, et al. Biochemical characterization of stage-specific isoforms of aspartate aminotransferases from *Trypanosoma cruzi* and *Trypanosoma brucei*. Mol Biochem Parasitol. 2008;161:12–20. doi:10.1016/j.molbiopara.2008.05.005
6. Loureiro I, Faria J, Clayton C, Ribeiro SM, Roy N, Santarem N, et al. Knockdown of asparagine synthetase A renders *Trypanosoma brucei* auxotrophic to asparagine. PLoS Negl Trop Dis 2013; 7:e2578. doi:10.1371/journal.pntd.0002578
7. Marie-Pierre H. Amino acid transporters in *Trypanosoma brucei brucei*. 2001. (Thesis (PhD)). Glasgow: University of Glasgow.
8. Steiger RF, Opperdoes FR, Bontemps J. Subcellular fractionation of *Trypanosoma brucei* bloodstream forms with special reference to hydrolases. Eur J Biochem 1980; 105:163–175. doi:10.1111/j.1432-1033.1980.tb04486.x
9. Marciano D, Maugeri DA, Cazzulo JJ, Nowicki C. Functional characterization of stage-specific aminotransferases from trypanosomatids. Mol Biochem Parasitol. 2009; 166:172–182. doi:10.1016/S0733-8619(03)00096-3
10. Duszenko M, Ferguson MA, Lamont GS, Rifkin MR, Cross GA. Cysteine eliminates the feeder cell requirement for cultivation of *Trypanosoma brucei* bloodstream forms in vitro. J Exp Med 1985;162:1256–63. doi:10.1017/CBO9781107415324.004
11. Southworth GC, Read CP. Absorption of some amino acids by the haemoflagellate, *Trypanosoma gambiense*. Comp Biochem Physiol A Physiol. 1972; 41:905–911. doi:10.1016/0300-9629(72)90354-4
12. Kaminsky R, Nickel B, Holý A. Arrest of *Trypanosoma brucei rhodesiense* and *T. brucei brucei* in the S-phase of the cell cycle by (S)-9-(3-hydroxy-2-phosphonylmethoxypropyl)adenine ((S)-HPMPA). Mol Biochem Parasitol. 1998; 93:91–100. doi:10.1016/S0166-6851(98)00023-1
13. Manchola NC, Rapado LN, Barisón MJ, Silber AM. Biochemical characterization of branched chain amino acids uptake in *Trypanosoma cruzi*. J Eukaryot Microbiol. 2015; c:1–10. doi:10.1111/jeu.12278
14. Hasne MP, Barrett MP. Transport of methionine in *Trypanosoma brucei brucei*. Mol Biochem Parasitol. 2000; 111:299–307. doi:10.1016/S0166-6851(00)00321-2
