# Supplementary material for: How much (ATP) does it cost to build a trypanosome? A theoretical study on the quantity of ATP needed to maintain and duplicate a bloodstream-form Trypanosoma brucei cell
Source: PLoS Pathog. 2023 Jul 27;19(7):e1011522. doi: 10.1371/journal.ppat.1011522 (PMC10409291; doi:10.1371/journal.ppat.1011522)
Supplement: S7 Table — (PDF) [file ppat.1011522.s007.pdf]

Supplementary Table S7. Calculations of synthesis flux for amino acids that can be produced from nutrients in CMM medium and their ATP flux

AA	Mol (%)	AA per cell	Synthesis flux (AA/cell cycle. cell)	ATP cost	Flux (ATP/cell cycle. cell)	Flux (fmol ATP/cell cycle. cell)
**Ala**	13.9	2.78×10^{-1}	5.25×10^{-2}	-2	-1.05×10^{-1}	-1.74×10^{-10}
**Asp**	7.4	1.48×10^{-1}	2.79×10^{-2}	-1	-2.79×10^{-2}	-4.64×10^{-11}
**Asn**	7.4	1.48×10^{-1}	2.79×10^{-2}	0	0	0
**Glu**	20	4.00×10^{-1}	7.55×10^{-2}	-1	-7.55×10^{-2}	-1.25×10^{-10}
