# Supplementary material for: How much (ATP) does it cost to build a trypanosome? A theoretical study on the quantity of ATP needed to maintain and duplicate a bloodstream-form Trypanosoma brucei cell
Source: PLoS Pathog. 2023 Jul 27;19(7):e1011522. doi: 10.1371/journal.ppat.1011522 (PMC10409291; doi:10.1371/journal.ppat.1011522)
Supplement: S1 Text — (PDF) [file ppat.1011522.s008.pdf]

Supplementary Text 1: Biosynthesis of amino acids

When BSF *T. brucei* are proliferating in rich medium all amino acids are taken up from the environment, the situation we addressed in our calculations in the main text of this paper. In contrast, in the minimal CMM medium only glutamine and cysteine are available and imported by the parasites (S1 Table) [1], while other amino acids necessary for cell proliferation can be produced from glucose, glutamine and cysteine. When amino acids are taken up, it occurs by dedicated transporters (reviewed by [2]). In the case of *in vitro* cultured BSF, the source of free amino acids can be the serum added to the medium. Alternatively, amino acids can be obtained from serum proteins, such as lipoproteins (taken up via receptor-mediated transporter) or albumin (taken up by fluid-phase endocytosis or specific importer proteins), followed by proteolysis [3–5] In order to estimate the energy demand of synthesizing amino acids, we calculated the production and expenditure of ATP in these biosynthetic processes from glutamine and cysteine. To this end, we searched the literature for characterized enzymes and the *T. brucei* genome database [6] for putative enzymes that can catalyze the production of different amino acids from these sources (S6 Table).

As amino acids are not consumed by BSF trypanosomes for ATP synthesis (reviewed in [2]), we assumed here that their use is predominantly for protein synthesis, while use for other purposes (such as post-translational modifications or the biosynthesis of metabolites that are present in small quantities) is likely relatively minor and therefore not considered in this work. For estimating the synthesis requirements for each amino acid, we used data from the amino acid composition of the proteome in BSFs [7]. Assuming BSFs have 1.35×10^8 proteins/cell and considering that its proteome will be doubled during the cell cycle, we calculated the synthesis flux for each amino acid per cell cycle (AA/cell cycle/cell). It is worth mentioning that in this calculation we are not considering proteome turnover, which has been calculated separately. For amino acids that can be produced from glutamine and pyruvate, fluxes for each amino acid per cell cycle were calculated, and net ATP consumption was obtained considering all reactions necessary to synthesize them (S6 and S7 Tables). As an example, one alanine (Ala) is obtained from one glutamate (Glu) and one pyruvate (Pyr). Each molecule of glutamate is formed from one glutamine (Gln), which is available in the medium. Pyruvate is obtained at a 2:1 molar ratio from glucose taken up the medium. It is important to remark that, differently from the culture conditions used for our calculations in the main text (parasites proliferating in the complex HMI-9 medium), here we are assuming that parasites proliferating in the minimal medium CMM use

some glucose-derived pyruvate to synthesize other metabolites required for biomass. Therefore, there is no net ATP demand for the biosynthesis of alanine but a net ATP production:

0 (Glu + Pyr -> Ala, no ATP required)
1 (Gln -> Glu, produces 1 ATP)
1 (glucose -> Pyr, produces 1 ATP)
----
2 (total yield of two ATP molecules per molecule of Ala produced)

Detailed reactions describing ATP yield/consumption of other amino acids are summarized in S6 and S7 Tables. The specific case of the biosynthesis of methionine from cysteine was not considered, because, despite having experimental support based on C-tracing experiments [8], the pathway is not yet well understood and, therefore, it is not possible to calculate the ATP expenditure.

References

1. Creek DJ, Nijagal B, Kim DH, Rojas F, Matthews KR, Barrett MP. Metabolomics guides rational development of a simplified cell culture medium for drug screening against *Trypanosoma brucei*. Antimicrob Agents Chemother. 2013;57: 2768–2779. doi:10.1128/AAC.00044-13
2. Marchese L, Nascimento J, Damasceno F, Bringaud F, Michels P, Silber A. The uptake and metabolism of amino acids, and their unique role in the biology of pathogenic trypanosomatids. Pathogens. 2018;7: 36. doi:10.3390/pathogens7020036
3. Büntemeyer H, Lütkemeyer D, Lehmann J. Optimization of serum-free fermentation processes for antibody production. Cytotechnology. 1991;5: 57–67. doi:10.1007/BF00365534
4. Langreth SG, Balber AE. Protein uptake and digestion in bloodstream and culture forms of *Trypanosoma brucei*. J Protozool. 1975;22: 40–53. doi:10.1111/j.1550-7408.1975.tb00943.x
5. Steverding D, Stierhof YD, Fuchs H, Tauber R, Overath P. Transferrin-binding protein complex is the receptor for transferrin uptake in *Trypanosoma brucei*. Journal of Cell Biology. 1995;131: 1173–1182. doi:10.1083/jcb.131.5.1173
6. Aslett M, Aurrecoechea C, Berriman M, Brestelli J, Brunk BP, Carrington M, et al. TriTrypDB: a functional genomic resource for the Trypanosomatidae. Nucleic Acids Res. 2010;38: D457–D462. doi:10.1093/nar/gkp851
7. Williamson J, Desowitz RS. Chemical composition of trypanosomes .1. Protein, amino acid and sugar analysis. Exp Parasitol. 1961;11: 161-. doi:10.1016/0014-4894(61)90023-6
8. Johnston K, Kim DH, Kerkhoven EJ, Burchmore R, Barrett MP, Achcar F. Mapping the metabolism of five amino acids in bloodstream form *Trypanosoma brucei* using U-13C-labelled substrates and LC–MS. Biosci Rep. 2019;39: 1–17. doi:10.1042/BSR20181601
