# Supplementary material for: How much (ATP) does it cost to build a trypanosome? A theoretical study on the quantity of ATP needed to maintain and duplicate a bloodstream-form Trypanosoma brucei cell
Source: PLoS Pathog. 2023 Jul 27;19(7):e1011522. doi: 10.1371/journal.ppat.1011522 (PMC10409291; doi:10.1371/journal.ppat.1011522)
Supplement: S2 Text — (PDF) [file ppat.1011522.s009.pdf]

Supplementary Text 2: What kills the BSF *T. brucei*?

Glycolysis has been proposed as a highly promising target for developing new drugs against sleeping sickness because BSF *T. brucei* are entirely dependent on this metabolic process for their ATP supply. In addition, the pathway is organized in a unique manner, with most of its enzymes present in glycosomes, and most of their enzymes having structural and functional properties very different from their mammalian homolog, or exerting a different level of control on the flux (reviewed in [1,2]). Analysis of trypanosomes in which different enzymes or transporters involved in glucose catabolism have been knocked down or inhibited demonstrated that 30-50% reduction of the glycolytic flux was sufficient to arrest growth of trypanosomes with further reduction resulting in death [3]. Indeed, inhibitors could be developed for several glycolytic enzymes that inhibit growth of cultured trypanosomes with no or limited effect on cultured human cells. Phosphofructokinase (PFK)-specific inhibitors have been prepared that caused very fast killing of trypanosomes in culture and cured parasitemia in a mice model of the acute stage of sleeping sickness without any apparent toxicity on the animal [4]. A preliminary metabolomics study of trypanosomes exposed to sublethal doses of PFK inhibitor demonstrated a partial ATP drop within minutes after addition of the compound, as well as changes in the level of several glycolytic intermediates, followed, within an hour, by other changes of the cellular metabolome [5]. Many of these latter changes are most likely due to the drop in ATP level. The multiple effects on the metabolome render it difficult to identify the precise mode of killing, also with knowledge about the relative ATP cost of different metabolic processes as discussed in the main text of this paper. Also relevant will be other aspects, for example if an inhibited process is essential for the parasite's viability, and if so at which time scale, and whether some metabolites will accumulate to toxic levels. Possibly, death is the cumulative result of the simultaneous disturbance of multiple processes.

References

1. Barros-Alvarez X, Gualdron-Lopez M, Acosta H, Caceres AJ, Graminha MAS, Michels PAM, et al. Glycosomal targets for anti-trypanosomatid drug discovery. Curr Med Chem. 2014;21: 1679–1706. doi:10.2174/09298673113209990139
2. Haanstra JR, Gerding A, Dolga AM, Sorgdrager FJH, Buist-Homan M, du Toit F, et al. Targeting pathogen metabolism without collateral damage to the host. Sci Rep. 2017;7: 40406. doi:10.1038/srep40406
3. Haanstra JR, Kerkhoven EJ, van Tuijl A, Blits M, Wurst M, van Nuland R, et al. A domino effect in drug action: from metabolic assault towards parasite differentiation. Mol Microbiol. 2011;79: 94–108. doi:10.1111/j.1365-2958.2010.07435.x
4. McNae IW, Kinkead J, Malik D, Yen L-H, Walker MK, Swain C, et al. Fast acting allosteric phosphofructokinase inhibitors block trypanosome glycolysis and cure acute African trypanosomiasis in mice. Nat Commun. 2021;12: 1052. doi:10.1038/s41467-021-21273-6

5. Nare Z, Moses T, Burgess K, Schnaufer A, Walkinshaw MD, Michels PAM. Metabolic insights into phosphofructokinase inhibition in bloodstream-form trypanosomes. Front Cell Infect Microbiol. 2023;13. doi:10.3389/fcimb.2023.1129791
