# Supplementary material for: How much (ATP) does it cost to build a trypanosome? A theoretical study on the quantity of ATP needed to maintain and duplicate a bloodstream-form Trypanosoma brucei cell
Source: PLoS Pathog. 2023 Jul 27;19(7):e1011522. doi: 10.1371/journal.ppat.1011522 (PMC10409291; doi:10.1371/journal.ppat.1011522)
Supplement: S3 Text — (PDF) [file ppat.1011522.s010.pdf]

Supplementary Text 3: Processes for which ATP costs could not be estimated due to lack of quantitative data.

DNA repair
DNA polymerase proofreading
Epigenetic modifications
Post-replication kDNA (minicircles and maxicircles) supercoiling
Formation of Open Transcriptional Complexes for the RNA pol I and III dependent transcription
Transcriptional termination
Phosphorylation of RNA polymerase II

Maintenance cost for the mitochondrial transcriptome
Mitochondrial pre-mRNA processing and RNA editing
Maintenance of the plasma membrane potential
Processes associated to chaperonin activities
Post-translational modifications of proteins requiring ATP
Signal transduction
Passive H^+ leakage through:

- Mitochondrial inner membrane
- Plasma membrane
- Acidocalcisome membrane
- Membranes of endocytic vacuoles

ATP consumption by intracellular pumps, among others:

- Ca^{2+}-ATPases in the endoplasmic reticulum and acidocalcisomes
- V-H^+-ATPase and V-H^+-PPase in the acidocalcisomes

V-H^+-ATPase in the endocytic pathway
Adaptation to possible environmental osmolarity changes during *in vivo* growth
Actin polymerization
